# Oxidative damage contributes to bisphenol S-induced development block at 2-cell stage preimplantation embryos in mice through inhibiting of embryonic genome activation

**DOI:** 10.1038/s41598-023-36441-5

**Published:** 2023-06-07

**Authors:** Anfeng Ning, Nansong Xiao, Hu Wang, Chunyi Guan, Xu Ma, Hongfei Xia

**Affiliations:** 1 Reproductive and Genetic Center & NHC Key Laboratory of Reproductive Health Engineering Technology Research, National Research Institute for Family Planning (NRIFP), Beijing, 100081 China; 2 Graduate Schools, Peking Union Medical College & Chinese Academy of Medical Sciences, Beijing, 100730 China

**Keywords:** Cell biology, Developmental biology

## Abstract

Although bisphenol S (BPS), as a bisphenol A (BPA) substitute, has been widely used in the commodity, it is embryotoxic in recent experiments. Nowadays, it remains unclear how BPS affects preimplantation embryos. Here, my team investigated the effects of BPS on preimplantation embryos and the possible molecular mechanisms in mice. The results showed that 10^–6^ mol/L BPS exposure delayed the blastocysts stage, and exposure to 10^–4^ mol/L BPS induced 2-cell block in mice preimplantation embryos. A significant increase in reactive oxygen species (ROS) level and antioxidant enzyme genes *Sod1*, *Gpx1*, *Gpx6*, and *Prdx2* expression were shown, but the level of apoptosis was normal in 2-cell blocked embryos. Further experiments demonstrated that embryonic genome activation (EGA) specific genes *Hsp70.1* and *Hsc70* were significantly decreased, which implied that ROS and EGA activation have the potential to block 2-cell development. Antioxidant enzymes, including superoxide dismutase (SOD), coenzyme Q10 (CoQ10), and folic acid (FA) were used to further explore the roles of ROS and EGA in 2-cell block. Only 1200 U/mL SOD was found to alleviate the phenomenon of 2-cell block, reduce oxidative damage, and restore the expression of EGA-specific genes *Hsp70.1* and *Hsc70*. Conclusively, this study demonstrates for the first time that BPS can induce 2-cell block, which is mainly mediated by ROS aggregation and results in the failure of EGA activation.

## Introduction

Early studies on the toxicity of bisphenol A (BPA) have found that it may have adverse effects on the reproductive and development system, the immune system, and the brain, etc.^1,2^. In 2010, Canada first proposed a ban on the sale of baby products containing BPA. Bisphenol S (BPS) is one of the BPA derivatives, and its maple group replaces the propane group of BPA. Since BPS is more resistant to acidity, heat, and sunlight than BPA, it is popular in food, medical, and paper products where the use of BPA is limited^3^. Reports in human urine samples from 2000 to 2014^4^ showed an increase in BPS concentrations and a decrease in BPA concentrations, and the median concentration of BPS found in human serum and urine samples are approximately 10^–9^ mol/L^5,6^. In addition, the residual amount of BPS was also found in the emulsion test^7^. However, BPS is one of the main ubiquitous compounds released from plastics^8^, and its concentration ranged from 2 × 10^–7^ to 4 × 10^–5^ mol/L in commercial plastic bottled water in a 2022 report^9^. For those occupationally exposed to BPS, the exposure level was 21,804 ng/day, which was much higher than the general population exposure level of 291 ng/day^10^. It is easy to see that the impact of BPS has far exceeded people's expectations.

Unfortunately, subsequent studies have found that BPS is harmful to embryos. For example, exposure of adult female zebrafish to BPS ≥ 0.5 μg/L for 21 days significantly decreased the percentage of F1 embryo development^11^. Moreman et al.^12^ found that BPS caused acute poisoning of zebrafish embryos. Gyimah et al.^13^ also found that low concentrations of BPS caused impaired central nervous system development in zebrafish embryos. These results suggest that BPS has embryotoxicity, but the effect of BPS on mammalian preimplantation embryo development is still unclear.

In molecular studies, it has been found that BPS can induce oxidative stress and inflammatory response in mice macrophages^14^ and can also reduce the viability of human ovarian granulosa cells by increasing reactive oxygen species (ROS)^15^. Through literature review, ROS is also found to be associated with preimplantation embryos. When preimplantation embryos are affected by adverse external factors, their ROS content is higher than the physiologically required value. The antioxidant system can eliminate the excess ROS in the embryo by regulating antioxidant enzymes and antioxidants, thereby maintaining the normal development of the preimplantation embryos^16^. Nevertheless, if the antioxidant capacity of the embryos is not sufficient to antagonize excessive ROS, it will hurt the preimplantation embryos with oxidative propensity^17^. The accumulation of ROS caused by oxidative stress can lead to developmental retardation, block, and apoptosis of preimplantation embryos^18^.

In the field of preimplantation embryonic development molecular mechanisms, it is found that the occurrence of 2-cell block is correlated with ROS level and embryonic genome activation (EGA). For example, Shi et al.^19^ found that ROS was significantly reduced, and EGA-specific genes were significantly increased in embryos in which melatonin relieved 2-cell block.

**Figure 1:**
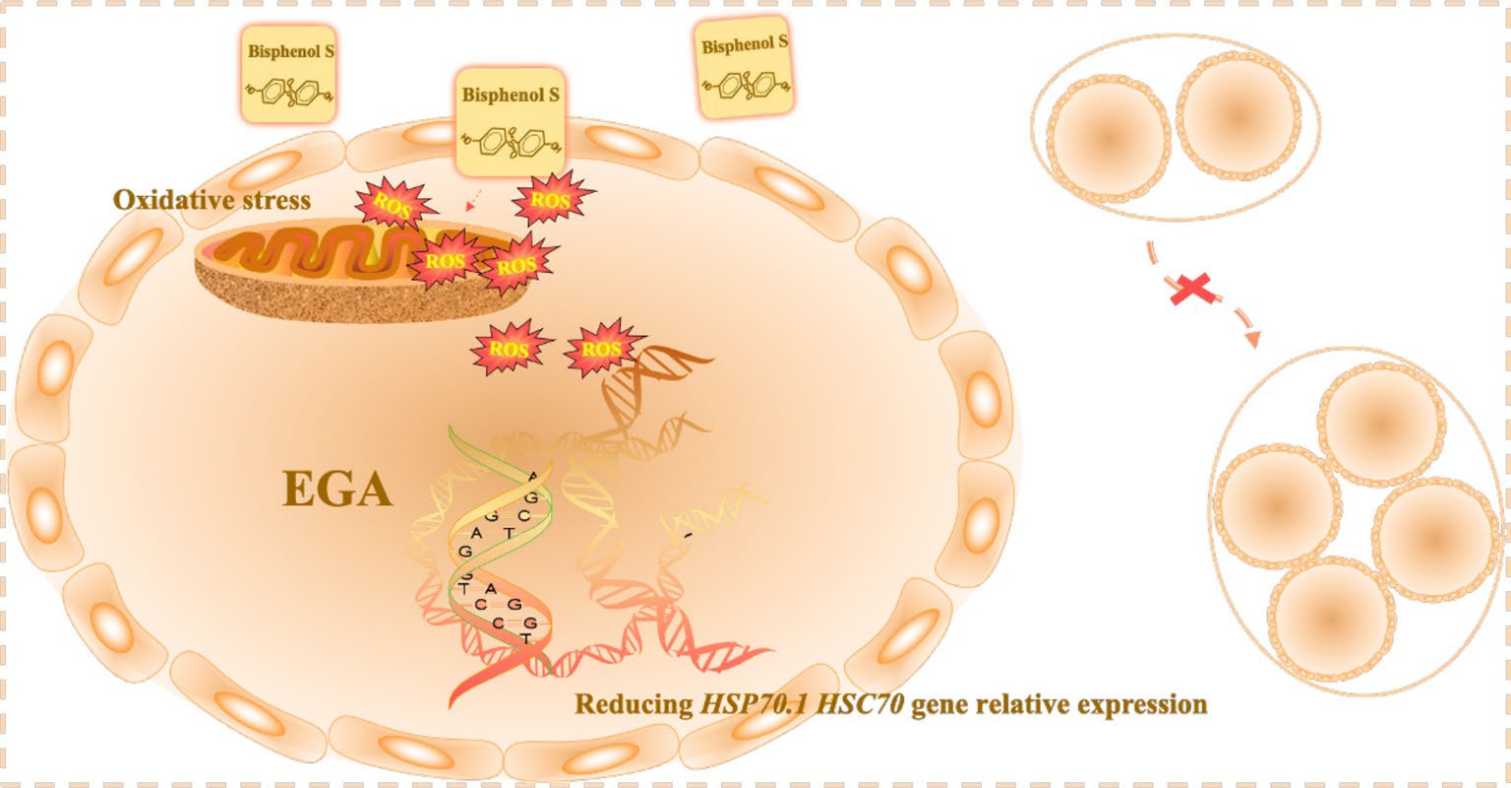
BPS-induced development block at 2-cell stage preimplantation embryos (Created using PowerPoint).

To better explore the effects of BPS on mice preimplantation embryos development and its molecular mechanisms, my team applied in-vitro fertilization (IVF) to control the BPS concentration around the preimplantation embryos or the processing time, to accurately elucidate the effects of BPS on the preimplantation embryos and verify the molecular mechanisms of preimplantation embryos development induced by BPS. These results not only help to reveal the embryotoxicity of BPS on the preimplantation embryos, but also provide a valuable reference for pregnant women and infants to choose dairy products rationally (Fig. 1).

## Materials and methods

### Animals

CD-1 (ICR) wild-type female mice (6–9 weeks) and male mice (10–13 weeks) were purchased from Weitong Lihua Bio-technology Co., Ltd (Beijing, China). All animals were kept in a specific pathogen-free facility with a 12 h/12 h light/dark cycle and were supplied with food and water ad libitum. All experimental procedures involving animals were approved and implemented following the guidelines outlined by the Institutional Animal Care and Laboratory Animal Welfare Ethics Committee of the National Research Institute for Family Planning.

### Embryo collection and culture

Before IVF, each female mouse was given an intraperitoneal injection of 7 U PMSG (Sansheng Bio-technology Co., Ningbo, China) and 8 U HCG (Sansheng Biotechnology Co., LTD., Ningbo, China). On the day of IVF, cumulus-oocyte complexes (COCs) taken from female mice oviduct and 2.5 μL (2.5 × 10^8^/mL) semen taken from male mice epididymis were placed into a culture medium (Sage In-Vitro Fertilization, Inc., Trumbull, CT, USA), and then put them in an incubator with 5% CO_2_ at 37 °C for 5 h, to undergo 2-cell, 4-cell, morula, and blastocyst stages.

For the dose–response study, zygotes were separately placed in Quinns Advantage Cleavage Media (Sage In-Vitro Fertilization, Inc.) containing 0 (setting control group), 10^–9^, 10^–8^, 10^–7^, 10^–6^, 10^–5^, and 10^–4^ mol/L BPS (Sigma-Aldrich, St. Louis, MO, USA) to incubate for 96 h. BPS was dissolved in DMSO to 10^–1^ mol/L, and then continued to dissolve BPS to the required concentration using culture medium. Thus, the highest concentration of DMSO (10^–3^ mol/L) in this experiment was selected as the sham group.

For the time response study, we selected 10^–4^ mol/L BPS with significant damage effect in the dose–response study. Zygotes were separately placed in Quinns Advantage Cleavage Media (Sage In-Vitro Fertilization, Inc.) containing10^–4^ mol/L BPS (Sigma-Aldrich, St. Louis, MO, USA), during 0, 3, 6, 9, 12, 24, and 48 h. In the experiment, we terminated the exposure time of BPS by continuing to culture embryos using normal medium.

In the antioxidant studies, zygotes were cultured in BPS with 0, 300, 600, 900, and 1200 Units/mL Superoxide dismutase (SOD) (Yuanye Bio-technology Co., Shanghai, China), 0, 10^–8^, 10^–7^, and 10^–6^ mol/L coenzyme Q10 (CoQ10) (Sigma-Aldrich, St. Louis, MO, USA), or 0, 4, 20, and 40 mg/L folic acid (FA) (Sigma-Aldrich, St. Louis, MO, USA).

### Mitochondrial distribution assay

Before preimplantation embryos were fixed in PBS with 4% paraformaldehyde for 15 min and then permeabilized in PBS with 0.5% Triton X-100 for 20 min at room temperature, mitochondrial distribution studies were undertaken using a Mito-tracker Red CMXRos kit according to the manufacturer's instructions (Biyuntian Bio-technology Co., Shanghai, China). After being washed three times in PBS, preimplantation embryos were placed in Mounting Medium With DAPI—Aqueous, Fluoroshield (DAPI) (Abcam, Cambridge, USA) for anti-quenching and nuclear staining. Signal intensities for A568 (red fluorescence) Ex/Em: 542/702 nm were detected under a confocal laser scanning microscope (CLSM) (ZEISS LSM 510 META; Carl Zeiss, Oberkochen, Germany).

### DNA extraction, qPCR analysis

Total DNA was isolated using a tissue/cell genomic DNA Extraction Kit according to the manufacturer's instructions (Jinbaite Bio-technology Co., Beijing, China). For qPCR, quantitative determination of *ND1* (For, 5′-GCCGTAGCCCAAACAATTTC-3′; Rev, 5′-CAGGCTGGCAGAAGTAATCATA-3′) DNA expression level was performed on a CFX96 (Bio-Rad) using Genious 2× SYBR Green Fast Qpcr Mix (Thermo Fisher Scientific, Sunnyvale, CA, USA) with *H2afz* (For, 5′-ATTGCTGGTGGTGGTATGTC-3′; Rev, 5′-GCACCCTGTACTGAAATCTCTT-3′) DNA used as an endogenous control. For the data, before using ΔΔCT methods^20^, the raw threshold cycle (CT) value was analyzed by BIO CFX96 software. The result was determined by 2^−ΔΔCt^ expressed as the relative mitochondrial DNA (mtDNA).

### RNA extraction, reverse transcription, and real-time PCR analysis

Total RNA was isolated, as described previously (Rio et al.^21^). The complementary DNA (cDNA) was synthesized using cDNA reverse transcription kit (TaKaRa, Japan) and real-time PCR was performed on a CFX96 (Bio-Rad) using Genious 2× SYBR Green Fast Qpcr Mix (Thermo Fisher Scientific) using *Sod1* (For, 5′-GGTTCCACGTCCATCAGTATG-3′; Rev, 5′-GTCTCCAACATGCCTCTCTTC-3′), *Gpx1* (For, 5′-CCTCTAAATTTGCACGGAGAAAC-3′; Rev, 5′-GAAAGGCATCGGGAATGGA-3′), *Gpx6* (For, 5′-TGAAGGCTGAGAGCAGAAAC-3′; Rev, 5′-AGGAAGGCTGGTAGGATATGA-3′), *Prdx2* (For, 5′- CCACCTGGAATCTGGTGAATAG-3′; Rev, 5′-CACAGACTTACACTGTCCCTAATAC-3′), *Hsp70.1* (For, 5′- CAGTAGCCTGGGAAGACATATAG-3′; Rev, 5′-TGTAGTACACAGTGCCAAGAC-3′), *Hsc70* (For, 5′-ACTCCTCTTTCCCTTGGTATTG-3′; Rev, 5′-GTCAGAGTAGGTGGTGAAAGTC-3′). The data analysis was performed using CFX96 software (Bio-Rad). *H2afz* (For, 5′-GGGAAGAAAGGACAACAGAAGA-3′; Rev, 5′-TCCACTGGAATCACCAACAC-3′) was used as an internal control, and results were determined by 2^−ΔΔCt^ expressed as the relative messenger RNA (mRNA).

### ROS assay

After preimplantation embryos were fixed with 4% paraformaldehyde for 30 min and then permeabilized with 0.5% Triton-X-100 for 40 min at room temperature, ROS studies were determined using a ROS Assay Kit according to the manufacturer's instructions (Biyuntian Bio-technology Co., Shanghai, China). After three washes in PBS, preimplantation embryos were placed in DAPI for anti-quenching and nuclear staining. Signal intensities for A488 (green fluorescence) Ex/Em: 493/630 nm were detected under a CLSM.

### Mitochondrial membrane potential assay

After preimplantation embryos were fixed with 4% paraformaldehyde for 15 min and then permeabilized with 0.5% Triton-X-100 for 20 min at room temperature, mitochondrial membrane potential studies were undertaken using a mitochondrial membrane potential assay kit with JC-1 according to the manufacturer's instruction (Biyuntian Bio-technology Co., Shanghai, China). After being washed three times in PBS, preimplantation embryos were placed in DAPI for anti-quenching and nuclear staining. Signal intensities for FITC (green fluorescence) Ex/Em: 494/562 nm were detected under a CLSM.

### Apoptosis determination

After preimplantation embryos were fixed with 4% paraformaldehyde for 30 min and then permeabilized with 0.5% Triton-X-100 for 40 min at room temperature, apoptosis was determined by TUNEL assay using a riboAPO One-Step TUNEL Apoptosis Kit (Red) according to the manufacturer's instruction (Ribo Bio-technology Co., Guangzhou, China). After being washed three times in PBS, preimplantation embryos were placed in DAPI for anti-quenching and nuclear staining. Signal intensities for A568 (red fluorescence) Ex/Em: 568/712 nm were detected under a CLSM.

### Statistical analysis

In this experiment, GraphPad Prism (Mac version 9.0.0, GraphPad Software, San Diego, California USA) was used for all data statistics. There were no less than three groups of experimental data, and the data results were expressed as mean ± SD. The independent sample *t*-test was used for the comparison of the mean values of the two groups, and one-way ANOVA was used for the comparison of multiple groups. It was believed that there was a significant difference in data when* P* < 0.05 (* or # represents *P* < 0.05, ** or ## represents *P* < 0.01).

## Results

### 10^–4^ mol/L BPS exposure induced 2-cell block in preimplantation embryos in mice

**Figure 2:**
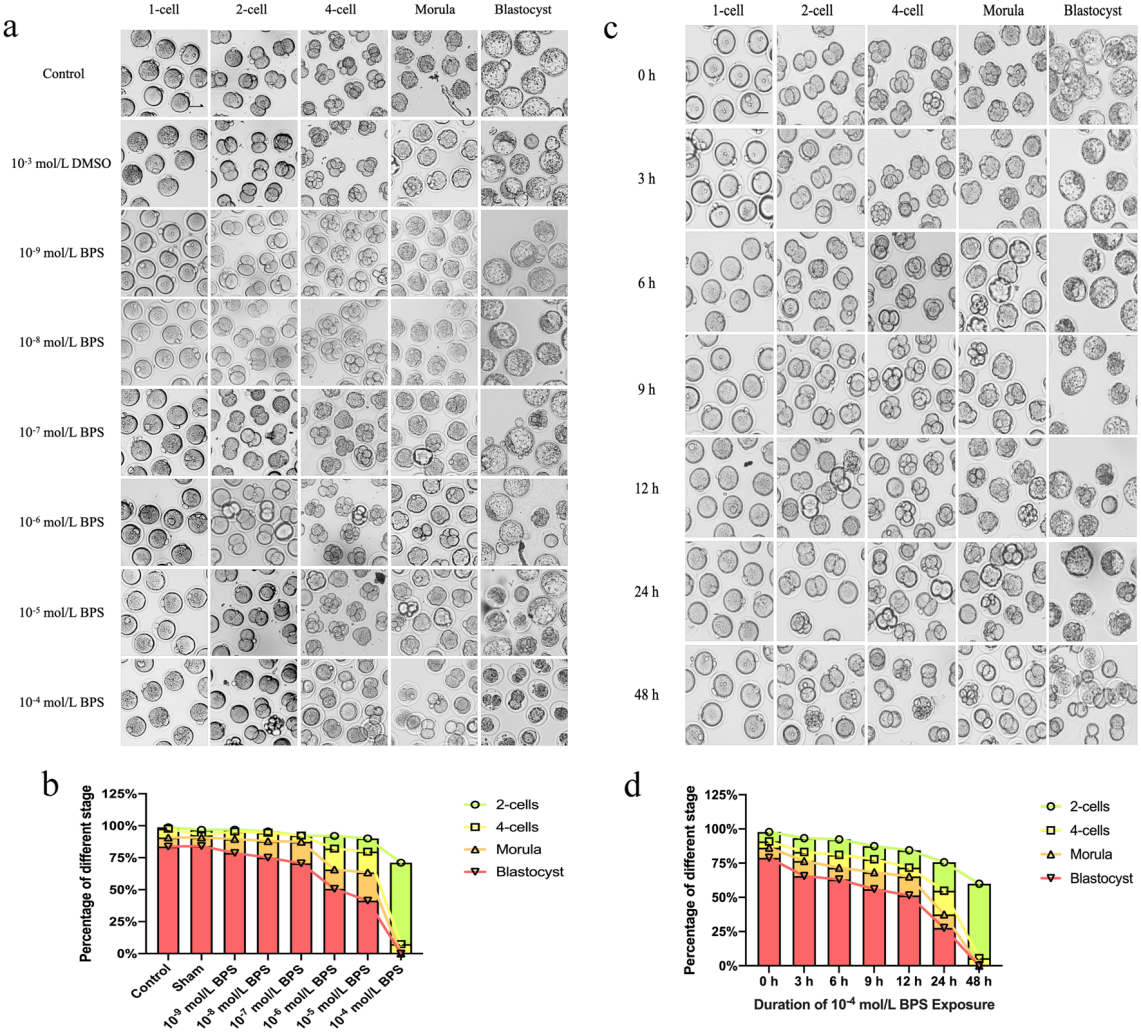
Effect of BPS exposure on preimplantation embryos development. Morphological images of preimplantation embryos treated by BPS (**a**) at different concentrations or (**c**) for different durations. BPS caused damage to preimplantation embryos in a (**b**) dose- and (**d**) time-dependent manner. Scale bar: 50 μm. Created using CLSM and GraphPad Prism.

To detect the effect of BPS on the developmental potency of preimplantation embryos, zygotes were treated with 10^–9^, 10^–8^, 10^–7^, 10^–6^, 10^–5^ and 10^–4^ mol/L BPS, and cultured to blastocyst. The embryos treated with nothing served as the control group. The embryos treated with 10^–3^ mol/L DMSO were selected as the sham group. The percentages of the cleavage, morula and blastocyst formation were not significantly different among control, sham, 10^–9^, 10^–8^, and 10^–7^ BPS-treated groups (Fig. 2a). 10^–6^ mol/L BPS exposure significantly reduced the percentages of the morula (*P* < 0.05) and blastocyst formation (*P* < 0.01). The percentages of the cleavage to the 4-cell stage (*P* < 0.01), and the development to the morula (*P* < 0.01) or blastocyst stage (*P* < 0.01) were substantially diminished by 10^–5^ mol/L BPS exposure. The percentage of the cleavage of 2-cell to 4-cell stage was less than 5% of embryos, and no embryos developed into morula and blastocyst stage in the 10^–4^ mol/L BPS group (Table 1). These results indicated that BPS destroyed the developmental potency of preimplantation embryos in a dose-dependent manner (Fig. 2b). and caused embryos to block at the 2–4 cell stage by 10^–4^ mol/L BPS treatment.

**Table 1:** Summary of the development of embryos exposed to BPS at different concentrations during the preimplantation period.

Treatment	N. of 1-cell embryos (0 h)	N. of 2-cell embryos (24 h, %)	N. of 4-cell embryos (48 h, %)	N. of morula (72 h, %)	N. of blastocyst (96 h, %)
Control	96	95 (99.0)	94 (97.9)	87 (90.6)	80 (83.3)
Sham	109	105 (96.3)	100 (91.7)	97 (89.0)	89 (81.7)
10^–9^ mol/L BPS	111	108 (97.3)	106 (95.5)	100 (90.1)	88 (79.3)
10^–8^ mol/L BPS	136	131 (96.3)	129 (94.9)	119 (87.5)	103 (75.7)
10^–7^ mol/L BPS	98	94 (95.9)	93 (94.9)	85 (86.7)	68 (69.4)
10^–6^ mol/L BPS	93	87 (93.5)	81 (87.1)	69 (74.2)*	53 (57.0)**
10^–5^ mol/L BPS	98	89 (90.8)	79 (80.6)**	66 (67.3)**	46 (46.9)**
10^–4^ mol/L BPS	114	76 (66.7)**	3 (2.6)**	0 (0.0)**	0 (0.0)**

Number and percentage of preimplantation embryos that developed to 2-cell, 4-cell, morula, and blastocyst stage in medium containing 0, 10^–9^, 10^–8^, 10^–7^, 10^–6^, 10^–5^, and 10^–4^ mol/L BPS.

Compared with the control group, *p < 0.05, **p < 0.01 (n ≥ 3). ANOVA, analysis of variance.

To analyze the effect of 10^–4^ mol/L BPS exposure time on the developmental potency of preimplantation embryos, zygotes were treated with 10^–4^ mol/L BPS for 0, 3, 6, 9, 12, 24, and 48 h (Fig. 2c) and then continued to be cultured in normal medium for up to 96 h. Compared to the control group (0 h group), the percentage of blastocyst formation was significantly decreased by 10^–4^ mol/L BPS exposure for 3 h (*P* < 0.05). The percentages of both morula and blastocyst formation were dramatically reduced by 10^–4^ mol/L BPS exposure for 6 h (*P* < 0.01). After BPS exposure time for 9 h, in addition to the significant reduction of the percentages of the morula and blastocyst formation, some embryos were blocked in 2-cell stage. The percentage of 2-cell block was 10.8% (*P* < 0.05), 12.6% (*P* < 0.01), 25% (*P* < 0.01), and 51.4% (*P* < 0.01) of embryos respectively at 9, 12, 24, and 48 h by BPS exposure. When the BPS exposure time reached 48 h, no embryos developed to the morula and blastocyst stage. The percentage of the cleavage zygotes to 2-cell stage was markedly reduced after 12 h of BPS exposure. The percentages of the blocking at 12, 24, and 48 h of BPS exposure were 15.8% (*P* < 0.05), 26.7% (*P* < 0.01), and 42.9% (*P* < 0.01) respectively (Table 2). These findings show that the percentage of 2-cell blocks gradually increased with the extension of exposure time by 10^–4^ mol/L BPS exposure (Fig. 2d), and the development of 2-cell embryos was almost completely blocked at 48 h of 10^–4^ mol/L BPS exposure.

**Table 2:** Summary of the development of embryos exposed to BPS for different durations during the preimplantation period.

Treatment	N. of 1-cell embryos (0 h)	N. of 2-cell embryos (24 h, %)	N. of 4-cell embryos (48 h, %)	N. of morula (72 h, %)	N. of blastocyst (96 h, %)
0 h	85	83 (97.6%)	77 (90.6%)	73 (85.9%)	67 (78.8%)
3 h	92	86 (93.5%)	77 (83.7%)	71 (77.2%)	61 (66.3%)*
6 h	118	109 (92.4%)	96 (81.4%)	85 (72.0%)**	75 (63.6%)**
9 h	92	81 (88.0%)	71 (77.2%)*	63 (68.5%)**	51 (55.4%)**
12 h	95	80 (84.2%)*	68 (71.6%)**	62 (65.3%)**	49 (51.6%)**
24 h	60	44 (73.3%)**	32 (53.3%)**	23 (38.3%)**	17 (28.3%)**
48 h	70	40 (57.1%)**	4 (5.7%)**	0 (0.0%)**	0 (0.0%)**

Number and percentage of preimplantation embryos that developed to 2-cell, 4-cell, morula, and blastocyst stage exposed to 10^–4^ mol/L BPS through 0, 3, 6, 9, 12, 24 and 48 h.

Compared with the control group, *p < 0.05, **p < 0.01 (n ≥ 3). ANOVA, analysis of variance.

### Oxidative damage is induced by BPS exposure in mice 2-cell block embryos

**Figure 3:**
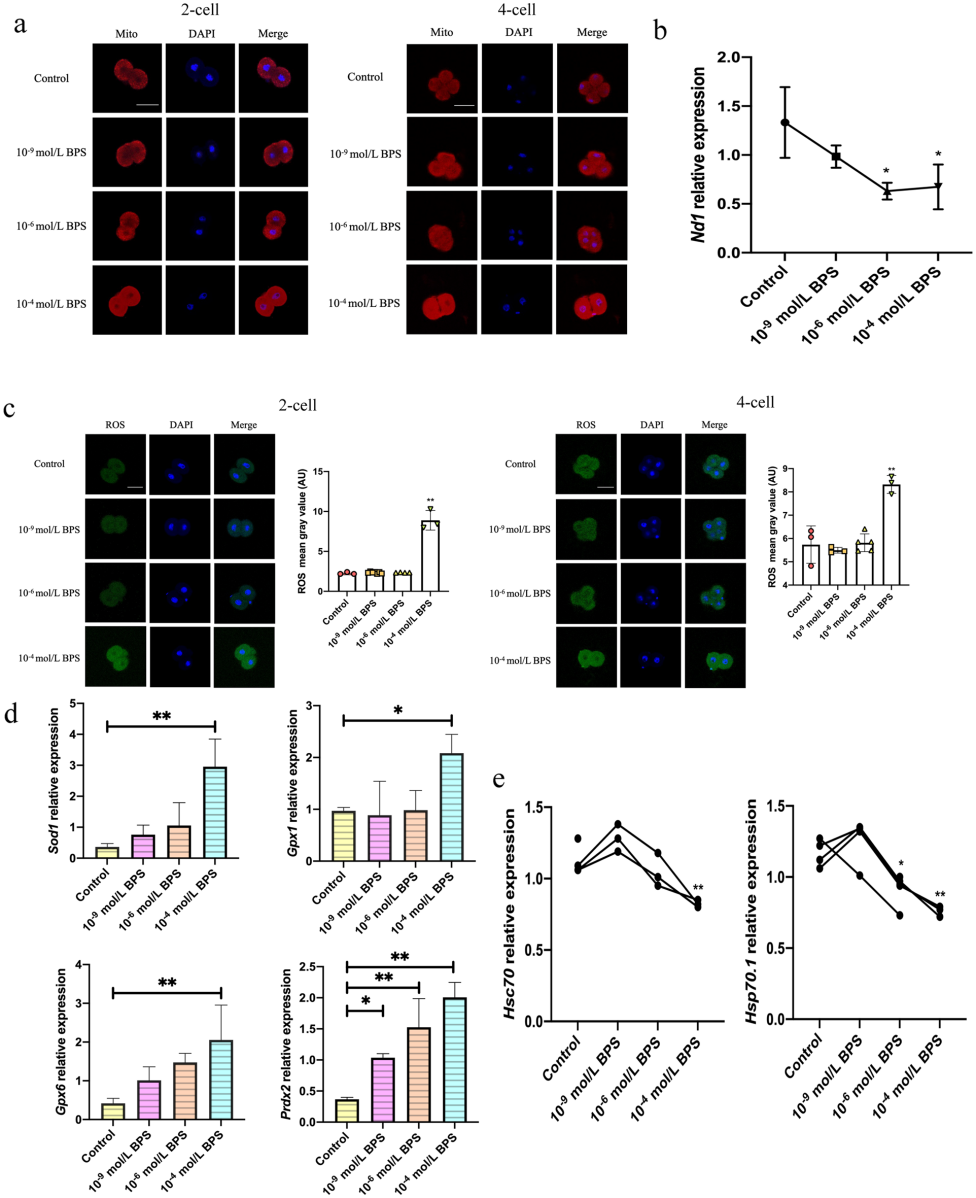
Oxidative damage and expression of EGA-specific genes decline by BPS exposure. (**a**) Mitochondrial distribution at 2- and 4-cell stage by Mito-Tracker Red CMXRos. (**b**) mtDNA (*ND1*) relative copy number at 2-cell to 4-cell transition stage by qPCR. (**c**) ROS levels at 2- and 4-cell stage by DCFH-DA. (**d**) Antioxidant enzyme genes *Sod1*, *Gpx1*, *Gpx6*, *Prdx2* and (**e**) EGA-specific genes *Hsp70.1*, *Hsc70* relative expressions at 2-cell to 4-cell transition stage by qPCR. Immunostaining of Mito-Tracker Red CMXRos is red in (**a**), immunostaining of DCFH-DA is green in (**c**) and nuclear stain DAPI is blue. Scale bar: 50 μm. Mito: Mito-tracker Red CMXRos. Data are represented as mean ± SD in (**b**–**d**) and single value in (**e**) (n ≥ 3). Compared with the control group, *p < 0.05, **p < 0.01. ANOVA, analysis of variance. Created using CLSM and GraphPad Prism.

Oxidative stress has always been considered one of the important causes of embryonic development block^16^. To verify whether oxidative stress occurred in 2-cell block embryos, my team selected 2- and 4-cell embryos treated with 10^–4^ mol/L BPS. In addition, 10^–9^ mol/L is the median concentration of BPS in human serum, and 10^–6^ mol/L is the highest concentration of BPS which can reduce the percentage of the blastocyst significantly. Thus, the two concentrations also were chosen in this experiment. In the 10^–4^ mol/L BPS group, the fluorescence signal of mitochondria around the nucleus was obvious (Fig. 3a) in response to proliferative stimuli^22^. *ND1* relative copy number, which can represent mtDNA relative copy number as a mitochondrial-encoded gene^23^, was downgrade significantly (*P* < 0.05) (Fig. 3b). In addition, my team also found fluorescence signal of ROS remarkably strengthen (*P* < 0.01), and antioxidant enzyme genes *Sod1*, *Gpx1*, *Gpx6*, *and Prdx2* significantly increased at 2-cell to 4-cell transition stage (*P* < 0.05) (Fig. 3c,d). These results indicated that 10^–4^ mol/L BPS induced oxidative damage in preimplantation embryos without damage to antioxidant enzyme systems.

### Apoptosis is normal by BPS exposure in mice 2-cell block embryos

**Figure 4:**
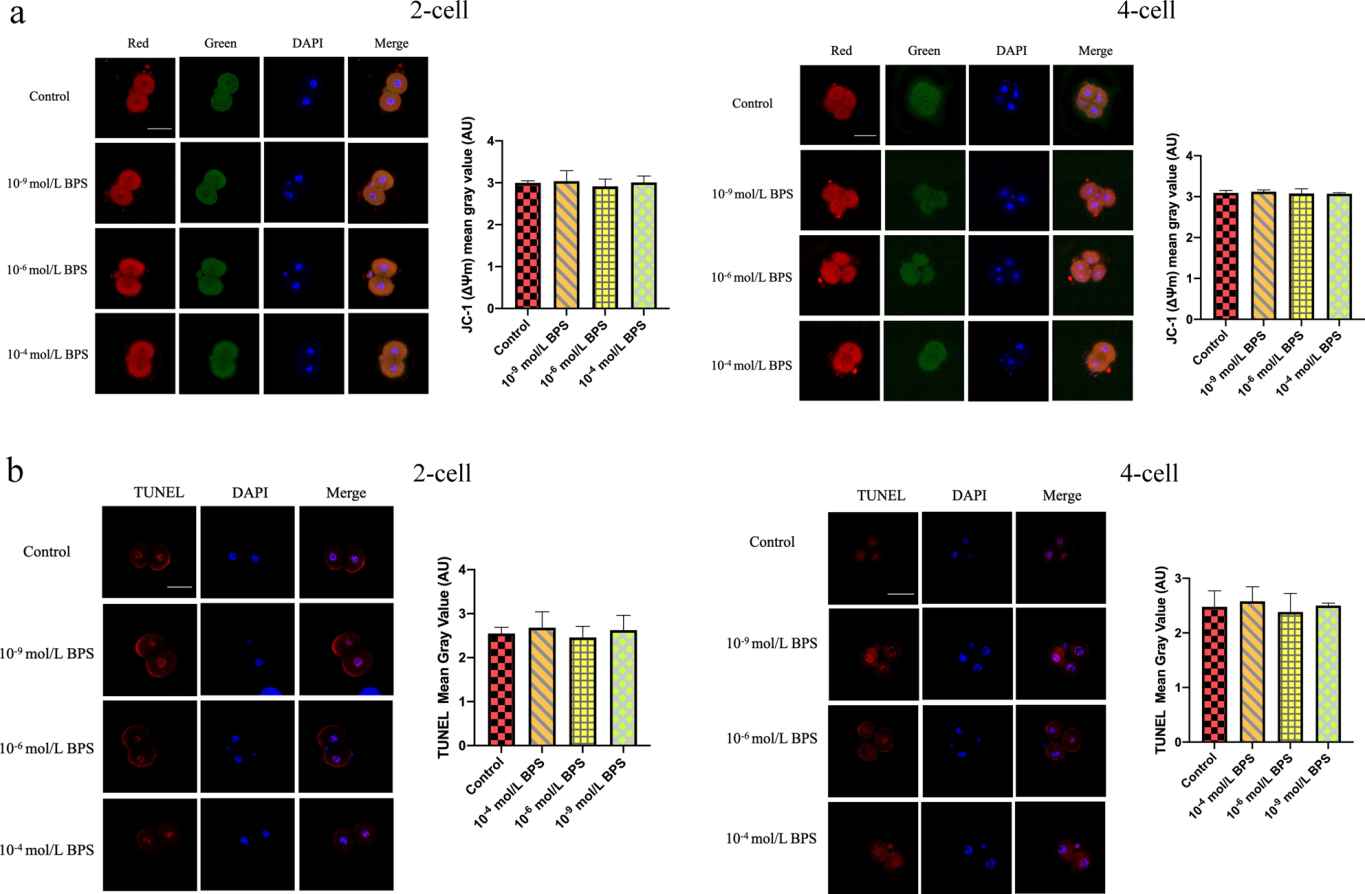
The level of apoptosis is normal by BPS exposure. The level of apoptosis was investigated by (**a**) membrane potential levels using JC-1 and (**b**) TUNEL using fluorescein-dUTP. In (**a**), ΔΨm (red fluorescence/green fluorescence) represents mitochondrial membrane level. In (**b**), immunostaining of fluorescein-dUTP is red. Nuclear stain DAPI is blue. Scale bar: 50 μm. Data are represented as mean ± SD (n ≥ 3). ANOVA, analysis of variance. Created using CLSM and GraphPad Prism.

To further investigate whether BPS causes the increase of apoptosis level, my team selected 2- and 4-cell preimplantation embryos exposed to 10^–9^, 10^–6^, and 10^–4^ mol/L BPS for mitochondrial membrane potential assay and apoptosis determination. The result showed that the 10^–9^, 10^–6^, and 10^–4^ mol/L groups had no significant difference as compared with the control groups (Fig. 4a), in both experiments (*P* > 0.05). The results showed that there was no significant difference between the BPS-induced group and the control group in apoptosis (Fig. 4b).

### BPS inhibits EGA activation in mice 2-cell block embryos

EGA, the transition from 2-cell to 4-cell stage, is the key event at 48 h of zygote development, at which zygotic genes are specifically expressed^24^. To verify whether BPS also causes EGA activation in 2-cell block preimplantation embryos, my team examined the relative expression of EGA-specific genes. It was found that *Hsp70.1* and *Hsc70* (Fig. 3e) were significantly downgraded in developed 48 h (2-cell to 4-cell transition stage) preimplantation embryos in 10^–4^ mol/L BPS group (*P* < 0.01). Accordingly, 10^–4^ mol/L BPS had the potential to inhibit EGA activation in the transition phase from 2-cell to 4-cell stage.

### SOD can antagonize 2-cell block in mice embryos by BPS exposure

**Figure 5:**
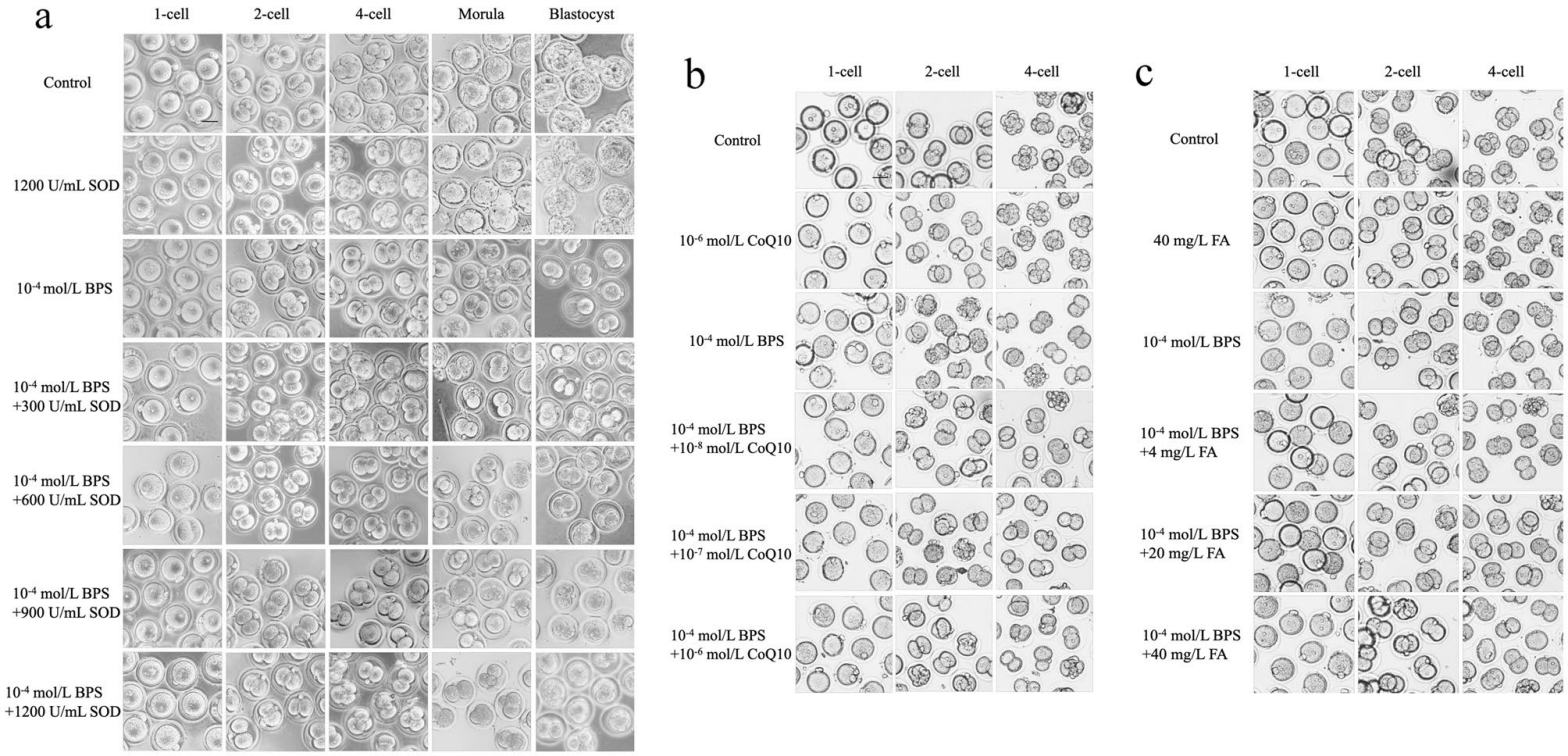
Effect of antioxidant enzymes on BPS-induced preimplantation embryos. Morphological images of BPS-induced preimplantation embryos in medium containing (**a**) SOD, (**b**) CoQ10, or (**c**) FA in different concentrations. Scale bar: 50 μm. Created using CLSM.

According to literature reports, antioxidant enzymes can enhance the production of ROS in embryos^16^. To antagonize the embryotoxicity of BPS in preimplantation embryos, SOD, FA and CoQ10 were added to the medium containing 10^–4^ mol/L BPS (Fig. 5a–c). The statistical results of the percentage of the development in 4 cell stages showed that 1200 U/ mL SOD can antagonize 2-cell block induced by 10^–4^ mol/L BPS (*P* < 0.01), but FA and CoQ10 cannot (*P* > 0.05) (Tables 3, 4, 5). Therefore, SOD had the potential to alleviate the embryotoxicity of BPS in preimplantation embryos.

**Table 3:** Summary of the development of embryos exposed to BPS and SOD during the preimplantation period.

Treatment	N. of 1-cell embryos (0 h)	N. of 2-cell embryos (24 h, %)	N. of 4-cell embryos (48 h, %)	N. of morula (72 h, %)	N. of blastocyst (96 h, %)
Control	100	98 (98.0)	91 (91.0)	90 (90.0)	82 (82.0)
1200 U/mL SOD	120	119 (99.2)	117 (97.5)	115 (95.8)	110 (91.7)*
10^–4^ mol/L BPS	97	77 (79.4)	3 (3.1)**	0 (0)**	0 (0)**
10^–4^ mol/L BPS + 300 U/mL SOD	76	59 (77.6)	4 (5.3)**	0 (0)**	0 (0)**
10^–4^ mol/L BPS + 600 U/mL SOD	94	75 (79.8)	7 (7.4)**	0 (0)**	0 (0)**
10^–4^ mol/L BPS + 900 U/mL SOD	117	90 (76.9)	18 (15.4)**	0 (0)**	0 (0)**
10^–4^ mol/L BPS + 1200 U/mL SOD	104	90 (86.5)	21 (20.2)**^##^	0 (0)**	0 (0)**

Number and percentage of preimplantation embryos induced by 10^–4^ mol/L BPS that developed to 2-cell, 4-cell, morula, and blastocyst stage in medium containing 300, 600, 900, and 1200 U/mL SOD.

Compared with the control group, *p < 0.05, **p < 0.01; compared with 10^–4^ mol/L BPS group, ^##^*p* < 0.01 (n ≥ 3). ANOVA, analysis of variance.

**Table 4:** Summary of the development of embryos exposed to BPS and CoQ10 during the preimplantation period.

Treatment	N. of 1-cell embryos (0 h)	N. of 2-cell embryos (24 h, %)	N. of 4-cell embryos (48 h, %)
Control	127	124 (97.6)	122 (96.1)
10^–6^ mol/L CoQ10	63	61 (96.8)	58 (92.1)
10^–4^ mol/L BPS	114	78 (68.4)**	4 (3.5)**
10^–4^ mol/L BPS + 10^–8^ mol/L CoQ10	60	40 (66.7)**	2 (3.3)**
10^–4^ mol/L BPS + 10^–7^ mol/L CoQ10	82	59 (72.0)**	3 (3.7)**
10^–4^ mol/L BPS + 10^–6^ mol/L CoQ10	60	47 (78.3)**	2 (3.3)**

Number and percentage of preimplantation embryos induced by 10^–4^ mol/L BPS that developed to 2-cell, 4-cell, morula, and blastocyst stage in medium containing 10^–8^, 10^–7^, and 10^–6^ mol/L CoQ10.

Compared with the control group, **p < 0.01 (n ≥ 3). ANOVA, analysis of variance.

**Table 5:** Summary of the development of embryos exposed to BPS and FA during the preimplantation period.

Treatment	N. of 1-cell embryos (0 h)	N. of 2-cell embryos (24 h, %)	N. of 4-cell embryos (48 h, %)
Control	119	116 (97.5)	112 (94.1)
40 mg/L FA	192	184 (95.8)	139 (72.4)**
10^–4^ mol/L BPS	97	72 (74.2)	2 (2.1)**
10^–4^ mol/L BPS + 4 mg/L FA	129	55 (42.6)	3 (2.3)**
10^–4^ mol/L BPS + 20 mg/L FA	140	62 (44.3)	4 (2.9)**
10^–4^ mol/L BPS + 40 mg/L FA	147	51 (35.4)	4 (2.7)**

Number and percentage of preimplantation embryos induced by 10^–4^ mol/L BPS that developed to 2-cell, 4-cell, morula, and blastocyst stage in medium containing 4, 20, and 40 mg/L FA.

Compared with the control group, **p < 0.01 (n ≥ 3). ANOVA, analysis of variance.

### SOD can alleviate BPS-induced oxidative damage in preimplantation embryos

**Figure 6:**
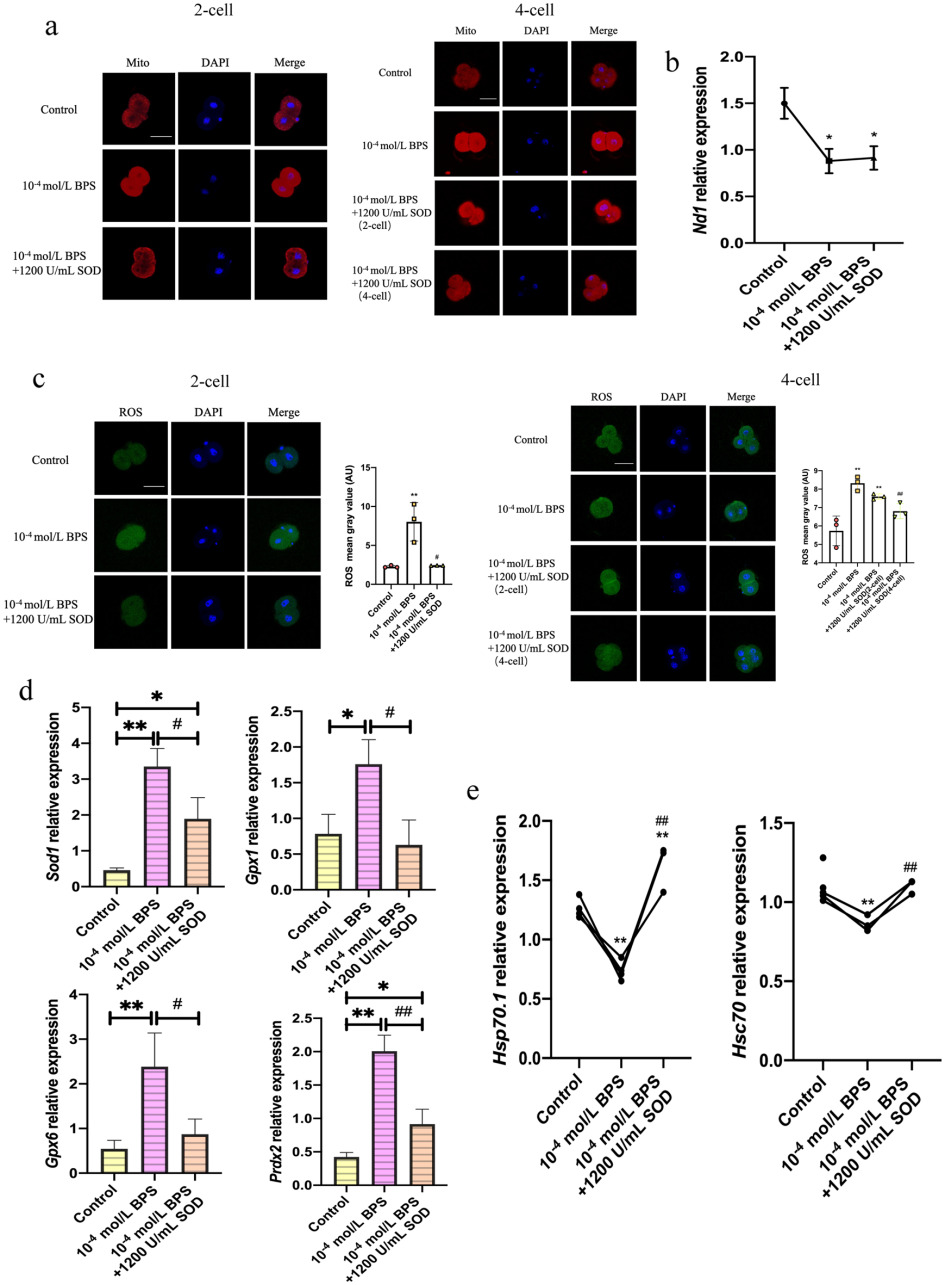
SOD can reduce oxidative damage and restore EGA-specific genes expression induced by BPS. (**a**) Mitochondrial distribution at 2- and 4-cell stage by Mito-Tracker Red CMXRos. (**b**) mtDNA (*ND1*) relative copy number at 2-cell to 4-cell transition stage by qPCR. (**c**) ROS levels at 2- and 4-cell stage by DCFH-DA. (**d**) Antioxidant enzyme genes *Sod1*, *Gpx1*, *Gpx6*, *Prdx2* and (**e**) EGA-specific genes *Hsp70.1*, *Hsc70* relative expression at 2-cell to 4-cell transition stage by qPCR. Scale bar: 50 μm. Data are represented as mean ± SD in (**b**), (**c**), (**d**) and single value in (**e**) (n ≥ 3). Compared with the control group, *p < 0.05, **p < 0.01. ANOVA, analysis of variance. Compared with 10^–4^ mol/L BPS group, ^#^*p* < 0.05, ^##^*p* < 0.01. *T* test, student's *t* test. Created using CLSM and GraphPad Prism.

In the 10^–4^ mol/L BPS + 1200 U/mL SOD group, the fluorescence signal of mitochondria did not cluster around the nucleus (Fig. 6a), but the mtDNA relative copy number changed slightly (*P* > 0.05) (Fig. 6b). The fluorescence signal of ROS and antioxidant enzyme genes *Sod1*, *Gpx1*, *Gpx6*, and *Prdx2* were restored (*P* < 0.05) (Fig. 6c,d). Moreover, the relative expression of *Hsp70.1* and *Hsc70* were also significantly recovered (*P* < 0.05) (Fig. 6e). Taken together, these results indicated that 1200 U/mL SOD can alleviate 10^–4^ mol/L BPS-induced 2-cell block phenotype, reducing oxidation damage to EGA activation in BPS-induced 2-cell block of mice embryos.

## Discussion

According to a prospective study, there are microplastics in placentas, meconium, infant feces, breastmilk, and infant formula^25^. Moreover, according to the results of the estimated daily intake (EDI) of BPS in the population of 12 countries, EDI decreased with age, which means that infants were the group with the highest BPS exposure in all age groups^26^. Therefore, it is of great significance to study the embryotoxicity of BPS.

At present, there are few studies on the effect of BPS on preimplantation embryo development and its molecular mechanisms. In this study, IVF technology was used to observe the effects of BPS on preimplantation embryos before implantation in vitro. Experimental studies have shown that 10^–4^ mol/L BPS has an obvious 2-cell blocking effect on preimplantation embryos in mice, which is consistent with the damaging phenotype of preimplantation embryos caused by 50 and 100 µg/kg body weight per day intraperitoneally for 21 consecutive days of BPS in female mice^27^, and 10^–6^, 10^−5^ mol/L BPS can reduce the percentage of the preimplantation embryos’ development. In addition, the longer the exposure time, the greater the toxicity of BPS. It is worth noting that treatment with 10^–4^ mol/L BPS for 3 h still caused significant damage to preimplantation embryos, indicating that the embryotoxicity of 10^–4^ mol/L BPS is very strong, and a large amount of instantaneous exposure can also cause serious damage to preimplantation embryos.

According to the other experiments results, my team found that in the 10^–4^ mol/L BPS group, mitochondria were distributed and clustered around the nucleus in response to proliferative stimuli^22^, the relative copy number of mtDNA was decreased obviously, the ROS level was significantly increased, and antioxidant enzyme genes *Sod1*, *Gpx1*, *Gpx6*, and *Prdx2* relative expression were significantly increased. These results may indicate that BPS can cause oxidative stress but cannot disrupt the antioxidant enzyme system in preimplantation embryos. The reason may be that the existence of high ROS leads to the improvement of the antioxidant capacity of preimplantation embryos^17^, but even so, it is difficult to counteract excessive ROS. In addition, transcription of the embryonic genome is robustly activated at 2-cell to 4-cell transition stage. If EGA activation fails, the embryos will not be able to continue dividing and will undergo developmental block^25^. In this study, the expression levels of EGA-specific genes *Hsp70.1* and *Hsc70* genes were significantly decreased. Therefore, excessive ROS may affect the activation of EGA, resulting in 2-cell block. In addition, it has been reported that excessive ROS can also activate the occurrence of apoptotic pathways^28^. However, in this study, mitochondrial membrane potential, one of the markers of early apoptosis^29^, was found no significant changes in the results and further detection by TUNEL did not show significant changes. Effects of silver nanoparticles on the development of preimplantation embryos in mice experiments showed that 20 nm silver nanoparticles at the concentration of 2 μg/mL also induced 2-cell block of preimplantation embryos with abnormal apoptosis levels detected^30^. The comparison results showed that there was a difference between 10^–4^ mol/L BPS and 2 μg/mL 20 nm silver nanoparticles. 2-cell blocked preimplantation embryos induced by 2 μg/mL 20 nm silver nanoparticles showed obvious shrinkage at the 4-cell stage, while 2-cell blocked preimplantation embryos induced by 10^–4^ mol/L BPS showed obvious shrinkage at the blastocyst stage and maintained normal 2-cell morphology at the preimplantation stage. Therefore, 2-cell blocked preimplantation embryos may activate the apoptosis pathway at the blastocyst stage, rather than at 2-cell or 4-cell stage. In addition, Guerin et al. (2001) believed that exogenous antioxidant substances could compensate for the antioxidant effect beyond their antioxidant capacity and antagonize more ROS during oxidative stress in preimplantation embryos.

Therefore, to antagonize the embryotoxicity of 10^–4^ mol/L BPS, antioxidant enzymes SOD, CoQ10, and FA were selected for the study. The experimental results showed that 1200 U/mL SOD can effectively relieve 2-cell block, but CoQ10 and FA did not. This may be due to the low toxicity of CoQ10^31^ and FA^32^, and the fact that SOD, as an antioxidant enzyme, has a direct effect on oxidative damage. The following experiments were carried out to test whether adding 1200 U/mL SOD in a culture medium containing 10^–4^ mol/L BPS can also improve other phenotypes. Mitochondrial distribution and ROS levels improved, approaching those of the control group, especially as preimplantation embryos developed into 4 cells. And the mtDNA relative copy number showed an upward trend. In addition, the number of antioxidant enzyme genes *Sod1*, *Gpx1*, *Gpx6*, and *Prdx2* expression were significantly declining, which means that the added exogenous antioxidant enzymes can improve the preimplantation embryos' antioxidant capacity. And my team found that when EGA-specific gene expression levels were measured during 1200 U/mL SOD treatment, *Hsp70.1 and Hsc70* expression in preimplantation embryos induced by 10^–4^ mol/L BPS were recovered to varying degrees. Thus, there is a correlation between ROS and EGA-specific genes.

## Conclusions and limitations

To sum up, although we thought that oxidation damage has the potential to inhibit EGA activation in mice 2-cell block embryos induced by BPS, more evidence is needed to support it. The main reason is that the molecular mechanisms of EGA is not clear, so that there are few methods to verify the relationship between ROS and EGA. Anyway, we analyzed the embryotoxicity of BPS in preimplantation embryos in this study, which can provide relevant data and theoretical support for the evaluation of the safe use of BPS.

## Data Availability

The datasets used or analyzed during the current study are available from the corresponding author on reasonable request.
